# Revisiting the History and Biogeography of *Bactrocera oleae* and Other Olive-Feeding Fruit Flies in Africa and Asia

**DOI:** 10.3390/insects16010030

**Published:** 2024-12-31

**Authors:** Luis Teixeira da Costa, Marie-Claude Bon, Barbara van Asch

**Affiliations:** 1 Norwegian Entomological Society, 0318 Oslo, Norway; luisteixeiracosta@gmail.com; 2 USDA ARS, European Biological Control Laboratory, 34980 Montferrier sur Lez, France; mcbon@ars-ebcl.org; 3 Department of Genetics, Stellenbosch University, Private Bag X1, Matieland 7602, South Africa

**Keywords:** mitochondrial, phylogeography, Oleaceae, *Olea europaea*, olive fruit fly

## Abstract

*Bactrocera oleae* Rossi causes important agricultural losses in olive growing regions. Despite its economic relevance, the expansion history and biogeography of *B. oleae* and other olive-feeding fruit flies remain unclear. We used mitogenomic data of *Bactrocera* species from a broad geographic range to explore possible historical biogeographic patterns in *B. oleae* and other olive-feeding fruit flies. Our data suggest that (1) the transition from stenophagy on Oleaceae to oligophagy on *Olea* most likely occurred in Africa more than 6 million years ago (MYA), and (2) the subsequent transition to monophagy on *Olea europaea* took place in Asia or in Africa in the Early Pleistocene. Our results support equally the hypotheses that the ancestors of modern *B. oleae* underwent two waves of migration from Asia to Africa or that they zigzagged between Asia and Africa.

## Simple Summary

The olive fruit fly, *Bactrocera oleae*, is considered the most important pest of cultivated olives in most world regions, some of which (e.g., California) were invaded in the modern era. The place and time of the evolution of *B. oleae* and related fruit fly species capable of feeding on olives is a matter of scientific interest that has remained unclear. Based on new genetic data generated from a diverse sample of olive fruit flies, we suggest that the ancestors of *B. oleae* started feeding on the genus *Olea* in Africa more than 6 million years ago, and later specialized on the *Olea europaea* group about 4 million years ago; however, it remains unclear whether this specialization occurred in Asia or in Africa.

## 1. Introduction

The olive fruit fly, *Bactrocera oleae* Rossi (Diptera: Tephritidae), is an oligophagous insect responsible for damage to cultivated olives (*Olea europaea* subsp. *europaea* L.). Agricultural losses due to *B. oleae* have been historically high in the Mediterranean Basin and the Middle East, where infestations were reported over 2000 years ago by Pliny the Elder [1]. *Bactrocera oleae* has been recorded as an agricultural pest in all world regions where olives are commercially cultivated except Australia and South America. The modern expansion of the *B. oleae* to North America (1998, California) [2] and Iran [3] and its rapid dissemination upon arrival further evidences its invasive potential [4,5]. The species is currently categorized by the European and Mediterranean Plant Protection Organization (EPPO) as a quarantine pest in California and Hawaii in the USA and Mexico, recommended for regulation as a quarantine pest in the A1 list (pests exotic to the region) in Iran and Bahrain, and in the A2 list (pests known in the region) in Canada and Europe.

The genus *Bactrocera* comprises about 460 species, most of them native to the Asia-Pacific region and using a wide range of hosts. *Bactrocera oleae* is one of the few species that is not native to the Asia-Pacific and that is able to utilize *Olea europaea* L., the olive complex currently composed of six subspecies and considered to have been the primary contributor to the cultivated olive [6]. *Bactrocera oleae* is not restricted to cultivated olives but also utilizes a diversity of wild *O. europaea.* The *O. europaea* complex extends across a vast geographic area spanning from southern and eastern Africa to the Mediterranean and stretching eastwards across southwest Asia to the Sino-Himalayan region [7]. *Olea europaea* subsp. *cuspidata* (Wall. ex G. Don) Cif. is one of the most widespread species, with major areas of natural distribution in southern and eastern Africa where it is found from South Africa northwards to Eritrea, and into the Middle East with occurrences in the Arabian Peninsula. The major Asian center of *O. e.* subsp. *cuspidata* is northern India, Afghanistan, Pakistan, and Kashmir, from which the species has reached the easternmost limit of its natural distribution in China, particularly the drier parts of the Yunnan and Sichuan provinces [8]. *Bactrocera oleae* has been reported in several points along the distribution of *O. e.* subsp. *cuspidata*, although data are currently missing for vast geographic areas [9]. *Olea e.* subsp. *cuspidata* is also the host of *Bactrocera biguttula* and *Bactrocera munroi*, the other two recognized olive-feeding fruit fly species, native to sub-Saharan Africa and closely related to *B. oleae* [9,10,11]. 

Previous phylogeographic studies of *B. oleae* have revealed substantial evolutionary divergence among its populations across Africa and Asia, underscoring the role of geography and host diversity in shaping its genetic structure. For instance, analyses of mitogenomes have shown that the Asian clade of *B. oleae*, represented by populations from Pakistan, is basal to all other lineages [9,12]. Preliminary studies of mitochondrial sequences from specimens in China suggest an even earlier divergence, indicating that *B. oleae* may have originated in Asia before expanding westward [9]. These findings suggest that wild *O. europaea.* subspecies, such as *O. e.* subsp. *cuspidata*, may serve as important reservoirs of genetic diversity, potentially influencing the pest’s ability to exploit cultivated olives.

Furthermore, phylogeographic studies have provided evidence of distinct ecological interactions in the pest’s native and introduced ranges. For example, the high diversity of specialized parasitoid wasps associated with *B. oleae* in South Africa [10,13,14] points to a complex evolutionary history shaped by biotic interactions in its native range. This information is critical for understanding how such interactions may differ in regions where the pest has been introduced and contribute to its invasive success.

The assemblage of parasitoid wasps attacking *B. oleae* has been systematically surveyed across large part of the geographic range of the olive fruit fly and its plant hosts, from sub-Saharan Africa to Himalayan Asia and China [4,8,11,13,14,15,16,17,18]. Interestingly, the highest species diversity and abundance of specialized parasitoids was found in the Eastern and Western Cape provinces of South Africa [10,14,15]. Phylogenetic analyses based on complete mitogenomes have shown the Asian clade of *B. oleae*, represented by Pakistan, as sister group to all other *B. oleae* lineages [3,12]. Furthermore, preliminary analysis of short mitochondrial sequences of *Bactrocera* specimens from China, morphologically identified as *B. oleae* and collected from *O. e.* subsp. *cuspidata*, suggested that these diverged from the remaining *B. oleae* at an even earlier time point [8]. Based on complete mitogenomes, Teixeira da costa et al. (2019) [9] highlighted that *B. oleae* is nested within an Asian cluster of the genus *Bactrocera* but, due to the limited geographic range of the sequence data analyzed, the study was not informative as to where African *B. oleae* are nested within Asian *B. oleae*.

The present work explores possible phylogeographic patterns of olive fruit fly evolution using new and publicly available mitogenomic sequence data from a broad geographic range (southern and eastern Africa, Reunion, Pakistan, and China) to deepen our understanding of the evolutionary relationships of African and Asian *B. oleae* with the other olive-feeding fruit fly populations and congeners. By incorporating these data, we aim to elucidate evolutionary relationships that can inform pest management strategies and improve understanding of the biological and ecological factors that contribute to the economic significance of *B. oleae* by exploring hypotheses that may explain when and where specialization in *Olea* occurred.

## 2. Materials and Methods

### 2.1. Sampling

Olive-feeding fruit flies were sampled at various locations in Asia (China and Pakistan), Africa (Kenya, South Africa and Namibia), and the Indian Ocean (Reunion) (Table S1). Specimens collected by the European Biological Control Laboratory in China and in Reunion Island are in the process of being deposited at the Smithsonian National Museum of Natural History, USA. In South Africa and Namibia, adult specimens were recovered from infested olive fruits, as previously described [14]. Adult specimens from Kenya, Reunion, Pakistan, and China were obtained from earlier collections and identified by Ian White (The Natural History Museum, London, United Kingdom) [8]. All specimens were stored at −20 °C until DNA extraction using a standard phenol-chloroform method [19].

### 2.2. Mitogenome Sequencing, Assembly, and Annotation

Specimen B011 (South Africa) was sequenced using Sanger technology, as previously described [9]. The other seven specimens were sequenced at a later stage using the Ion GeneStudio™ S5™ Prime platform (ThermoFisher Scientific, Waltham, MA, USA), available at the Central Analytical Facilities of Stellenbosch University, South Africa. In brief, sequence libraries were prepared using the Ion Plus Fragment Library Kit™ (ThermoFisher Scientific, Waltham, MA, USA), according to the manufacturer’s protocol MAN0009847, REV K.0. The final, purified libraries were quantified using the IonLibrary TaqMan™ Quantification kit (ThermoFisher Scientific, Waltham, MA, USA) according to the protocol MAN0015802, REV C.0. The Ion Torrent reads were trimmed with a 30-base sliding-window at an average threshold of Q16. The remaining sequencing reads were then filtered for read length and reads shorter than 25 bases were removed from the dataset.

Assembly of the Ion Torrent reads was performed using the mitogenome of *B. oleae* (GenBank GU108466) as reference on Geneious Prime v2023.1.2 (https://www.geneious.com/; accessed 1 June 2024) under the “Map to reference” function with the low to medium sensitivity option and fine-tuning up to five iterations. The open reading frames of the expected 13 mitochondrial protein-coding genes (PCGs) were identified with Geneious Prime using the invertebrate mitochondrial genetic code. Transfer RNAs (tRNA) were predicted with ARWEN [20] (http://130.235.244.92/ARWEN/; accessed on 1 June 2024, under the composite metazoan mitochondrial genetic code. The most likely tRNA positions were manually identified by comparison to other *B. oleae* mitogenomes available on GenBank, as well as the position of ribosomal RNAs and the AT-rich region.

### 2.3. Phylogenetic Reconstruction

The phylogeny of the genus *Bactrocera* was estimated in the context of thirty-two species for which mitogenomes were publicly available, four newly sequenced *Bactrocera* (two *B. munroi,* and one specimen from China and one specimen from Reunion both morphologically identified as *B. oleae*), and seven outgroup species (one from the sub-family Tephritinae, two from the tribe Ceratitidini, two from the genus *Dacus*, and two from the genus *Zeugodacus*) (Table S1) [9,12,21,22,23,24,25,26,27,28,29,30,31,32,33,34,35,36,37]. When more than one mitogenome sequence for the same species was available on GenBank, we opted to include only the sequence deposited on the database as the NCBI reference for the species (i.e., NC_XXXXXX) except in two cases: the reference sequence for *B. minax* (NC_014402) contained errors, as previously reported [9] and, in the case of *B. rubigina*, we opted for using the sequence MT121270 because it is associated with the paper by Zhang et al. (2023) [37], as opposed to direct submission to GenBank without an associated publication. It should be noted that there is an inconsistency in the classification of the sequence with the Genbank accession MN883026: while the authors [30] consider it to be *B. cheni*, it appears as *B. tsuneonis* on GenBank. This discrepancy likely results from conflicting views regarding the taxonomic status of *B. cheni*, specifically whether *B. cheni* and *B. tsuneonis* should be considered the same or separate species [30]. Given the large divergence between MN883026 and other mitogenomic sequences deposited on GenBank as *B. tsuneonis*, we opted for treating MN883026 as a separate species (*B. cheni*). The phylogeny of olive-feeding fruit flies was reconstructed using all complete *B. oleae* and *B. biguttula* mitogenome sequences available on Genbank (*n* = 40) and eight new mitogenome sequences generated in the present study: four *B. oleae* (two from Pakistan and two from Southern Africa), two specimens collected in China and in Reunion Island and morphologically identified as *B. oleae*, and two *B. munroi* (Table S1).

Both the phylogeny of the genus *Bactrocera* (including 33 species) and the phylogeny of olive-feeding fruit flies (only *B. oleae*, *B. biguttula*, and *B. munroi*) were estimated by maximum likelihood (ML) and Bayesian inference (BI). For all trees, the 13 PCGs were aligned separately, and then concatenated to produce a single alignment. ML analyses (three independent runs per tree) were run on the IQ-TREE [38] webserver [39] with automatic detection of evolutionary model using 1000 ultrafast bootstrap replicates [40]. For BI, the alignments were partitioned into the three codon positions in MEGA7 [41], and phylogenetic analyses were conducted in BEAST [42] on the CIPRES portal [43] (both accessed in 1 August 2024) using separate GTR + G nucleotide substitution and lognormal relaxed clock models for each partition to model a single tree. Nucleotide substitution priors were set with a normal distribution with mean and standard distribution values set as equal to the ML values calculated using jModelTest [44] run on CIPRES. As there are no suitable calibration points for the olive fruit fly tree, molecular clock and age priors were transferred from other analyses [3,9] based on an olive fly tree calibrated using the relevant nodes from a Diptera tree that was produced using paleontological calibration points [12]; the results of the *Bactrocera* tree are estimated here in the case of the root age of the olive-feeding fruit fly tree. A Yule speciation process and a coalescent exponential growth were used to model the *Bactrocera* tree and the olive-feeding fruit fly tree, respectively. The trees were left unconstrained except for requirement of monophyly of Dacinae. Three separate runs of ten million steps were performed for each tree, with sampling every one thousand steps and burn-in of one million steps. Separate runs were combined using LogCombiner [45], and the final trees were generated with TreeAnnotator [45] and visualized using FigTree (http://tree.bio.ed.ac.uk/software/figtree/) and GIMP 2.10.34 (https://www.gimp.org/), all accessed on 1 August 2024).

## 3. Results and Discussion

**Figure 1:**
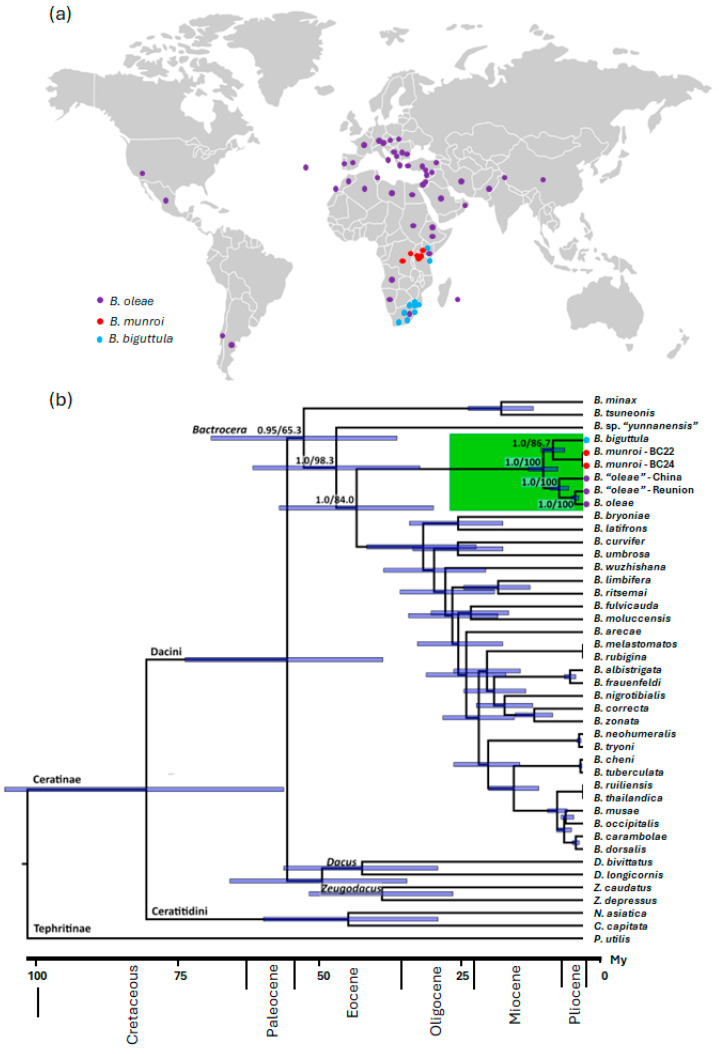
(**a**) Geographic distribution of *Bactrocera oleae*, *Bactrocera biguttula*, and *Bactrocera munroi*. The points were drawn based on data from the EPPO Global Database (accessed on 1 December 2024), the True Fruit Flies of the Afrotropical Region database of the Royal Museum for Central Africa, and the authors’ observations. (**b**) Time-calibrated phylogenetic tree of the genus *Bactrocera* based on new and publicly available mitogenome sequences (n = 36) with representatives of other Tephritidae species in the genera *Dacus*, *Zeugodacus*, *Neoceratitis*, *Ceratitis*, and *Procecidochares* as outgroup. The olive-feeding fruit fly clade, for which the present-day distribution is represented in (**a**), is highlighted in green, and all other species have their native range in the Asia-Pacific region. The scale bar represents time in millions of years ago, and the blue bars across nodes represent the 95% confidence intervals around the mean of the estimated split dates. Nodal support is shown as posterior probability and bootstrap on the nodes relevant for the phylogenetic relationships among olive-feeding fruit flies.

**Figure 2:**
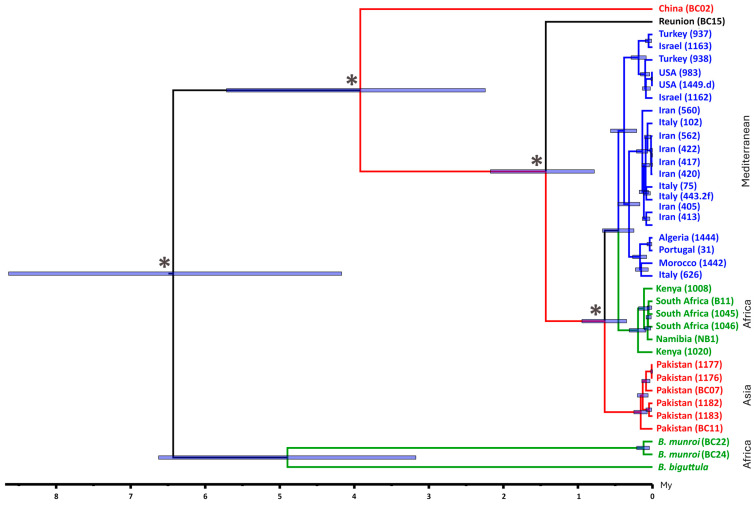
Time-calibrated phylogenetic tree of olive-feeding fruit flies displaying the divergence between *Bactrocera biguttula* and *Bactrocera munroi* (both native to Africa, shown at the bottom of the tree), and the *Bactrocera oleae* branch (presently widespread) based on new and publicly available mitogenome sequences (n = 36). The scale bar represents time in million years. Asterisks represent nodes with full support (posterior probability = 1; bootstrap = 100). Red branches—Asian lineages (Pakistan); green branches—African lineages (*B. munroi* and *B. biguttula*, and *B. oleae* from Kenya, South Africa and Namibia); blue branches—Mediterranean lineages Turkey, Israel, USA, Iran, Italy, Algeria, Portugal, Morocco); grey branch—lineages of undetermined origin (Reunion).

The BI and the ML trees for the genus *Bactrocera* (Figure 1) and the BI and ML trees for the olive-feeding fruit flies (Figure 2) have identical topologies, with high nodal sport. The mean node age of the genus *Bactrocera* is estimated to be about 49 (63.4–13.5) MYA, and it separates the Sino-Indian *B. minax* and the Sino-Japanese *B. tsuneonis* from the other species (Figure 1). An undescribed species from southern China (*Bactrocera* sp. “yunannensis”) appears split from the other *Bactrocera* species, in line with a previous suggestion for an origin of *Bactrocera* in eastern Asia [37]. However, the overlap between the node age estimates does not allow for robust conclusions based on node order. 

The tree for the genus *Bactrocera* also shows that olive-feeding fruit flies—i.e., species specializing in the genus *Olea* and using *Olea europaea* as a host—form an early diverging clade that split from most other *Bactrocera* included in this analysis (except *B. minax*, *B. tsuneonis*, and *B.* sp. “*yunannensis*”) approximately 40 MYA (Figure 1). This estimate is in general agreement with the more comprehensive fruit fly phylogeny presented in Starkie et al. (2022) [46], where *B. oleae* clusters with *B. minax* and *B. tsuneonis* (and several other species) in a clade splitting from most other *Bactrocera* soon after the divergences of *Hemizeugodacus* and *Apodacus*. Therefore, it seems that the oldest branches of the *Bactrocera* phylogeny contain species native to the Pacific region, the younger branches include species native to the Pacific region and/or Asia, and the olive-feeding fruit flies are nested among them. Overall, and for the time being, these results do not invalidate the hypothesis of an Asian origin for the ancestors of extant African and Mediterranean olive-feeding fruit flies.

But when and where did the specialization in *Olea* occur? Our results allow insights with regards to when the *Bactrocera* specializations—first in *Olea* and then in *O. europaea*—took place. The crown age of the olive-feeding fruit fly clade, defined by the separation of *B. munroi* and *B. biguttula* from the rest, provides a minimum estimate for the specialization in *Olea* at about six million years ago (Figure 2). As for the second specialization, it is known that *B. oleae* is monophagous on *O. europaea*, while *B. munroi* and *B. biguttula* also use other *Olea* species (*O. welwitschii* and *O. woodiana*, respectively) as hosts [47]. Considering that the olive-feeding fruit flies from China (morphologically identified as *B. oleae*) were found only on *O. europaea* [8], this specialization must have occurred before the split between the olive-feeding fruit flies from China and bona fide *B. oleae*, estimated at about 4 million years ago. Interestingly, both this estimate and the estimated time of divergence of the olive-feeding fruit flies from Reunion and bona fide *B. oleae* (about 1.4 MYA) are larger than those for other pairs of recognized species, lending support to the notion that the olive-feeding fruit flies from China and from Reunion belong to two novel species, as previously hypothesized [8]. Conversely, we identified cases (e.g., *Bactrocera thailandica* and *Bactrocera ruiliensis*) where hitherto recognized species are estimated to have diverged more recently than individual specimens of *B. oleae* within a single Mediterranean clade (Figure 1 and Figure 2). Barring species misidentification, these results suggest that the status of some recognized *Bactrocera* species may have to be re-evaluated.

As for where the *Bactrocera* specializations occurred, the results of Starkie et al. (2022) [48] provide intriguing clues as the abovementioned clade containing *B. oleae* and *B. minax* also includes species in the subgenus *Parazeugodacus*, several of which are specialized in Oleaceae. This is relevant because—although multiple other *Bactrocera* species at various positions in the *Bactrocera* tree can use Oleaceae as host plants—all use plants in a wide variety of families. In contrast, at least four *Parazeugodacus* species are known to be specialized in Oleaceae, three of which use *Olea* species as hosts: *Bactrocera nigra* and *Bactrocera fulvifacies* use *Olea paniculata*, and *Bactrocera abbreviata* uses *Olea salicifolia*. Importantly, *B. nigra* and *B. abbreviata* are not exclusive on *Olea*, as both use *Chinonanthus ramiflorus* (another Oleaceae) as host. Nevertheless, it seems reasonable to speculate that the specialization in Oleaceae and the ability to use *Olea* as host were already present at an early stage in the evolutionary history of *Bactrocera*. Given that the geographic distribution of both *O. salicifolia* and *O. paniculata* extends westwards—in the case of *O. paniculata*, all the way to Pakistan—it would also be tempting to speculate that the process of specialization in *Olea*, potentially involving *O. paniculata* as host, also occurred in the Asia-Pacific region, with a later westward expansion. However, in a previous survey in India where large numbers of *O. paniculata* fruit were collected, no insects were recovered; therefore, no hard data are available to support that hypothesis.

**Figure 3:**
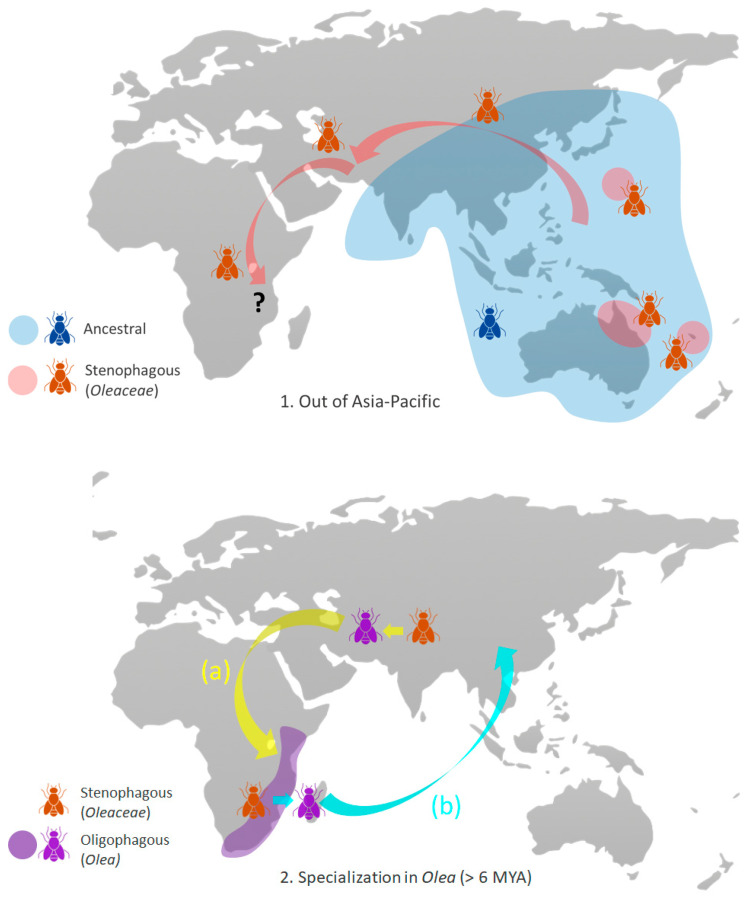

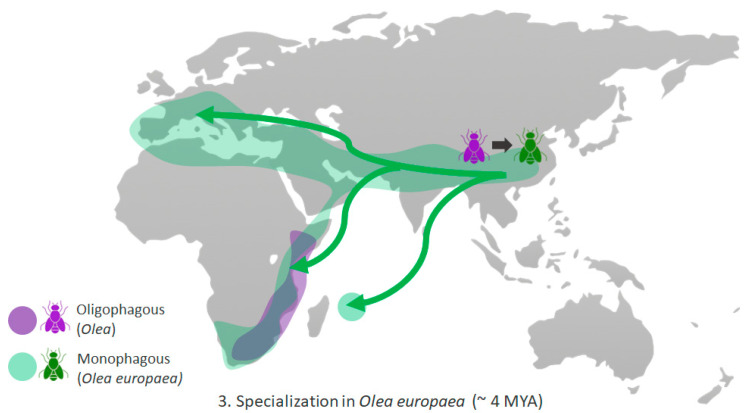
Hypotheses for the phylogenetic history of olive-feeding fruit flies: (**1**) Initial origin and dispersion of fruit flies stenophagous on Oleaceae. Dispersion of stenophagous fruit flies may have reached Africa, as indicated by the question mark. (**2**) Subsequent transition to oligophagy on *Olea* may have occurred in Asia (short yellow arrow) or in Africa (short turquoise arrow) in the Late Miocene (>6 MYA). In either case, these oligophagous olive fruit flies reached both continents (long arrows). The extant geographic range is depicted (purple). (**3**) Later, the transition to monophagy in *Olea europaea* occurred in Asia in the Early Pliocene (~4 MYA), with subsequent dispersion throughout the historic geographic range of the host. Arrows indicate the direction of dispersals, and shaded areas indicate approximate geographic ranges.

Due to the inclusion of olive-feeding fruit flies from China and from Reunion as well as additional specimens from Pakistan and southern Africa, our results provide a new perspective on where the specialization of *Bactrocera* in *O. europaea* occurred. Indeed, both the ML and the BI analyses strongly support *B. oleae* from Africa as sister clade to the Pakistan clade, both nested within a larger clade comprising an Asian lineage (from China), supporting the notion that specialization in *O. europaea* occurred in Asia (Figure 3). However, this hypothesis could be invalidated by additional data regarding the origin of Reunion’s population. Because Reunion is geographically considered part of Africa, it might be tempting to assume an African origin for its *O. europaea*-feeding populations. However, it is difficult to reconcile that notion with the absence of similar populations in the neighboring and much larger island of Madagascar. On the contrary, a monsoon driven spread of *B. oleae* (or ancestors of *B. oleae*) can easily be envisioned. In the absence of a definitive answer, we opted for not classifying the Reunion populations as being of Asian of African origin (Figure 2). Additionally, our phylogeny of olive-feeding fruit flies also suggests that populations of *B. oleae* in east Africa are older than their counterparts in southern Africa, in agreement with the notion of an invasion from Asia.

Could the biogeography of the *Olea* host offer contextualization for the evolution of olive fruit flies? The geographic origin of the genus *Olea* has not been deduced but an African origin for the subgenus *Olea* at 20–10 MYA was suggested [7]. *Olea europaea* split into *O. e.* subsp. *europaea* (found in the Mediterranean and in isolated regions of sub-Saharan Africa) and *O. e.* subsp. *cuspidata* (with two lineages: tropical Africa to Kenya/Ethiopia and Asia), which occurred in the Quaternary (6.1–4.4 MYA) [12]. The natural distribution of *O. europaea* species presently extends from South Africa to South Asia, although their distribution in some regions, including the area in China where our specimens were collected, is patchy [8]. These specimens appear in the most basal position in our phylogenetic reconstruction, followed by *B. oleae* found in Reunion. The *B. oleae* from Pakistan, which is closer to the “Mediterranean” and “African” clades than the China and Reunion lineages, was a well-differentiated group based on mtDNA and microsatellites in agreement with its classification as a distinct taxonomic subspecies or variety (*B. oleae* var. *asiatica*: Silvestri 1916) [49] and was later considered a relict population [12]. These results highlight the wide range of extant lineages of *B. oleae*, in line with a long-term presence of olive fruit flies across a vast geographic expansion following the historical distribution of *O. europaea*. The most common recent ancestor of the six *Olea* subspecies was present in Late Miocene to Early Pliocene (8.3–4.0 MYA) [6], in agreement our hypothesis of *Bactrocera* specialization in *Olea* at more than 6 MYA. The early divergence of the eastern and southern Africa lineage of *O.* subsp. *cuspidata* from the remaining lineages appears to have occurred at approximately 6.1 (8.3–4.0) MYA, at the time of the aridification of African midlatitudes [6,50], a notion that is supported by recent phylogenomic data [51]. The gene flow between Mediterranean/North African and sub-Saharan populations of *B. oleae* has likely been limited since the aridification processes that started in the late Miocene. Our phylogeny evidences the sister-clade relationship between the “Mediterranean” and the “African” *B. oleae*, which further diverged when populations in the Mediterranean co-evolved with *O. europaea* shifting from the wild host (*O. europaea* subsp. *europaea* var. *sylvestris*) to domesticated olives (*O. europaea* subsp. *europaea* var. *europaea*), a much richer food source, along with the expansion of agriculture in the Neolithic [12]. These populations must have gone through significant chemosensory and other behavioral adaptations since specialized fruit flies are finely tuned to locating their host for oviposition, which will be most interesting to study in the future.

Our results, and particularly the phylogeny of the olive-feeding fruit flies (Figure 2), are compatible with specialization on *Olea* having occurred in Africa or in Asia. The first hypothesis, mainly supported by the fact that so far oligophagous olive-feeding fruit flies have only been identified in Africa, would imply that the ancestors of *B. oleae* migrated out of Africa. The second hypothesis is that there were two waves of ancestral migration from Asia into Africa. These hypotheses may remain unconfirmed until genomic data on a wide range of *Bactrocera* species become available. Recently, genomic and transcriptomic data on 10 fruit fly species challenged the mitochondrial phylogenies in that *B. dorsalis* was recovered more closely related to *B. latifrons* than to *B. tryoni* [52]. Therefore, the phylogenetic relationships among the species within the “olive clade” we present here may also be challenged when genomic data become available. RAD-seq data were recently used for clarifying the well-known taxonomic confusion within the *B. dorsalis* complex, a group that contains several agricultural pests worldwide, many of which are under strict surveillance and control. The study revealed two new species, instances of introgression, and will have impact on records of host and distribution ranges and species identification protocols vital for regulating trade and establishing quarantine measures [53]. Mitochondrial data can only provide a limited perspective on complex micro- and macroevolutionary processes affecting species, and genomic data will be necessary for clarifying the origin and dispersal of fruit flies that feed on *Olea* and eventually understanding the genetic basis of this unique adaptation.

In summary, the data presently available support the following hypotheses for the evolution of olive-feeding fruit flies shown in Figure 3: (1) *Bactrocera* originated either in eastern Asia or the Pacific region and expanded west and southward throughout Asia and into Africa; among those *Bactrocera*, some were stenophagous specializing in Oleaceae; (2) Further specialization of *Bactrocera* in *Olea* hosts occurred either in Asia (a) or in Africa (b). In either case, their range expanded to include both continents; (3) In Asia, the oligophagous *Bactrocera* specialized in *O. europaea*, subsequently expanding west and southward into the Mediterranean and sub-Saharan Africa, following the historical distribution of *O. europaea*.

## Data Availability

The sequence data generated in this study was deposited on GenBank under the accession numbers PQ801847-PQ801854.

## Supplementary Materials

The following supporting information can be downloaded at: https://www.mdpi.com/article/10.3390/insects16010030/s1, Table S1: List of sequences used in the phylogenetic reconstructions. Table S2: Length of the individual 13 mitochondrial protein coding genes (PCGs), and their concatenation.
