# Supplementary material for: Revisiting the History and Biogeography of Bactrocera oleae and Other Olive-Feeding Fruit Flies in Africa and Asia
Source: Insects. 2024 Dec 31;16(1):30. doi: 10.3390/insects16010030 (PMC11766006; doi:10.3390/insects16010030)
Supplement: Supplementary file 1 [file insects-16-00030-s001.zip › insects-3289995-supplementary.pdf]

Revisiting the history and biogeography of *Bactrocera oleae* and other olive-feeding fruit flies in Africa and Asia

Teixeira da Costa L, Bon M-C, van Asch B

SUPLEMMENTARY TABLES

Table S1. List of new and publicly available mitogenome sequences used in this study for phylogenetic reconstruction of the genus *Bactrocera*, with other Tephritidae (*Dacus*, *Zeugodacus*, *Neoceratitis*, *Ceratitis* and *Procecidochares*) as outgroup. Question marks denote unknown or presumed geographic origin of the specimens sequenced in previous studies (n. a. – not applicable).

Species	World Region	Country	Genbank	Reference
Bactrocera albistrigata	Asia-Pacific (?)	Indonesia	MH374118	Unpublished
Bactrocera arecae	Asia	Malaysia	NC_028327	[32]
Bactrocera biguttula	Africa	South Africa	NC_042712	[9]
Bactrocera bryoniae	Pacific (?)	Papua New Guinea	NC_071745	[37]
Bactrocera carambolae	Asia	Japan	NC_009772	Unpublished
Bactrocera correcta	Asia	China	NC_018787	Unpublished
Bactrocera curvifera	Pacific (?)	Papua New Guinea	NC_071737	[37]
Bactrocera dorsalis	Asia	China	NC_008748	Unpublished
Bactrocera frauenfeldi	Pacific (?)	Papua New Guinea	NC_062138	[37]
Bactrocera fulvicauda	Pacific (?)	Papua New Guinea	NC_071738	[37]
Bactrocera latifrons	Asia	Malaysia	NC_029466	[33]
Bactrocera limbifera	Asia-Pacific (?)	Indonesia	NC_037722	[31]
Bactrocera melastomatos	Asia	Malaysia	NC_029467	[33]
Bactrocera minax	Asia	China	NC_014402	[37]

Bactrocera moluccensis	Pacific (?)	Papua New Guinea	NC_071739	[37]
Bactrocera munroi (BC22)	Africa	Kenya	PQ801847	This work
Bactrocera munroi (BC24)	Africa	Kenya	PQ801848	This work
Bactrocera musae	Pacific (?)	Papua New Guinea	MT121266	[37]
Bactrocera neohumeralis	Pacific (?)	Australia	NC_062139	[29]
Bactrocera nigrotibialis	Asia	India	NC_071741	[37]
Bactrocera occipitalis	Pacific (?)	Philippines	NC_071742	[37]
Bactrocera oleae	Mediterranean	Portugal	AY210702	[24]
Bactrocera oleae (presumed; BC15)	Indian Ocean	Reunion Island	PQ801850	This work
Bactrocera oleae (presumed; BC02)	Asia	China	PQ801849	This work
Bactrocera oleae (BC11)	Asia	Pakistan	PQ801853	This work
Bactrocera oleae (BC07)	Asia	Pakistan	PQ801852	This work
Bactrocera oleae (NB01)	Africa	Namibia	PQ801851	This work
Bactrocera oleae (BO11)	Africa	South Africa	PQ801854	This work
Bactrocera ritsemai	Asia-Pacific (?)	Indonesia	NC_037723	[26]
Bactrocera rubigina	Asia	China	NC_046521	[37]
Bactrocera ruiliensis	Asia	China	NC_046952	[25]
Bactrocera sp. 'yunnanensis'	Asia	China	OK127889	Unpublished
Bactrocera thailandica	?	China (?)	NC_053983	Unpublished
Bactrocera tryoni	Pacific (?)	Australia (?)	NC_014611	[12]
Bactrocera tsuneonis	?	China (?)	MN883026	[30]
Bactrocera tuberculata	Asia	China	NC_071743	[37]
Bactrocera umbrosa	Asia	Malaysia	NC_029468	[33]
Bactrocera wuzhishana	Asia	China	NC_071744	[37]
Bactrocera zonata	Asia	India	NC_027725	[21]
Ceratitis capitata	n. a.	n. a.	NC_000857	[27]
Dacus bivittatus	Africa	South Africa	NC_046468	[35]
Dacus longicornis	n. a.	n. a.	NC_032690	[23]
Neoceratitis asiatica	Asia	China	MF434829	[28]
Procecidochares utilis	Asia	China	NC_020463	Unpublished

Zeugodacus caudatus	Asia-Pacific	Indonesia	KT625492	[30]
Zeugodacus depressus	n. a.	n. a.	NC_071732	[37]

Table S2. Length of the individual 13 mitochondrial protein coding genes (PCGs), and their concatenation. The stop codons were removed before the concatenation was used in the phylogenetic reconstruction of the genus *Bactrocera*.

**Species**	**Sequence**	**ATP6**	**ATP8**	**COX1**	**COX2**	**COX3**	**CYTB**	**ND1**	**ND2**	**ND3**	**ND4**	**ND4L**	**ND5**	**ND6**	**All**
Bactrocera albistrigata	MH374118	678	162	1535	690	789	1135	940	1022	352	1341	297	1721	525	11187
Bactrocera arecae	NC_028327	678	162	1535	690	789	1137	940	1023	352	1341	297	1713	525	11182
Bactrocera biguttula	NC_042712	678	162	1535	690	789	1137	940	1023	354	1341	297	1721	525	11192
Bactrocera bryoniae	NC_071745	678	162	1534	690	789	1137	940	1023	354	1341	291	1713	525	11177
Bactrocera carambolae	NC_009772	678	162	1535	690	789	1137	940	1023	352	1341	297	1721	525	11190
Bactrocera correcta	NC_018787	678	162	1535	690	789	1137	940	1023	354	1341	297	1721	525	11192
Bactrocera curvifera	NC_071737	678	162	1535	690	789	1137	940	1023	352	1341	297	1720	525	11189
Bactrocera dorsalis	NC_008748	677	162	1535	690	789	1135	940	1023	352	1341	297	1720	524	11185
Bactrocera frauenfeldi	NC_062138	678	162	1535	690	789	1135	940	1022	354	1341	297	1720	525	11188
Bactrocera fulvicauda	NC_071738	678	162	1534	690	789	1137	940	1023	354	1341	297	1720	525	11190
Bactrocera latifrons	NC_029466	678	162	1535	690	789	1135	940	1023	352	1341	297	1717	525	11184
Bactrocera limbifera	NC_037722	678	162	1535	687	789	1137	940	1023	354	1341	297	1720	525	11188
Bactrocera melastomatos	NC_029467	678	162	1535	690	789	1135	940	1023	352	1341	297	1720	525	11187
Bactrocera minax	NC_014402	678	162	1535	687	792	1137	940	1023	352	1341	297	1717	525	11186
Bactrocera moluccensis	NC_071739	678	162	1535	690	789	1137	940	1023	354	1341	297	1720	525	11191
Bactrocera munroi (BC22)	PQ801847	677	162	1535	690	789	1137	940	1023	354	1341	297	1722	525	11192
Bactrocera munroi (BC24)	PQ801848	677	162	1535	690	789	1137	940	1023	354	1341	297	1722	525	11192
Bactrocera musae	MT121266	678	162	1535	690	789	1137	940	1023	354	1341	297	1720	525	11191
Bactrocera neohumeralis	NC_062139	678	162	1535	690	789	1135	940	1023	352	1341	297	1720	525	11187
Bactrocera nigrotibialis	NC_071741	678	162	1534	690	789	1137	937	1023	354	1341	297	1720	525	11187

Bactrocera occipitalis	NC_071742	678	162	1535	690	789	1137	940	1023	354	1341	297	1720	525	11191
Bactrocera oleae	AY210702	677	162	1535	690	789	1137	940	1023	354	1341	297	1719	525	11189
Bactrocera oleae China BC02 (presumed)	PQ801849	677	162	1535	690	789	1137	940	1023	354	1341	297	1719	524	11188
Bactrocera oleae Reunion BC15 (presumed)	PQ801850	677	162	1535	690	789	1137	940	1023	354	1341	297	1719	524	11188
Bactrocera oleae (BC11)	PQ801853	677	162	1535	690	789	1137	940	1023	354	1341	297	1719	524	11188
Bactrocera oleae (BC07)	PQ801852	677	162	1535	690	789	1137	940	1023	354	1341	297	1719	524	11188
Bactrocera oleae (NB01)	PQ801851	677	162	1535	690	789	1137	940	1023	354	1341	297	1719	524	11188
Bactrocera oleae (B011)	PQ801854	677	162	1535	690	789	1137	940	1023	354	1341	297	1719	524	11188
Bactrocera ritsemai	NC_037723	678	162	1535	687	789	1137	940	1023	354	1341	297	1720	525	11188
Bactrocera rubigina	NC_046521	675	153	1499	690	789	1137	940	1023	354	1341	297	1720	525	11143
Bactrocera ruiliensis	NC_046952	675	165	1498	690	789	1137	940	1023	354	1341	297	1720	525	11154
Bactrocera sp. 'yunnanensis'	OK127889	678	162	1534	690	789	1137	940	1023	354	1341	297	1722	525	11192
Bactrocera thailandica	NC_053983	678	162	1532	690	789	1137	940	1023	354	1341	297	1720	525	11188
Bactrocera tryoni	NC_014611	678	162	1535	690	789	1135	940	1023	352	1341	297	1720	525	11187
Bactrocera tsuneonis	NC_038164	678	162	1535	687	789	1135	939	1023	343	1341	297	1729	525	11183
Bactrocera tuberculata	NC_071743	678	162	1534	690	789	1137	937	1023	354	1341	297	1720	525	11187
Bactrocera umbrosa	NC_029468	678	162	1535	690	789	1135	940	1023	352	1341	297	1720	525	11187
Bactrocera wuzhishana	NC_071744	678	162	1535	690	789	1137	940	1023	354	1341	297	1720	525	11191
Bactrocera zonata	NC_027725	678	162	1535	690	789	1137	940	1023	352	1341	297	1721	525	11190
Ceratitis capitata	NC_000857	678	162	1535	687	789	1137	940	1023	354	1341	291	1720	525	11182
Dacus bivittatus	NC_046468	678	162	1534	690	789	1137	939	1023	354	1341	297	1717	525	11186
Dacus longicornis	NC_032690	678	162	1534	690	789	1135	940	1023	354	1341	297	1720	522	11185
Neoceratitis asiatica	MF434829	678	162	1535	687	789	1137	940	1023	354	1341	291	1720	525	11182
Procecidochares utilis	NC_020463	678	162	1534	692	789	1134	939	1023	354	1341	291	1729	525	11191
Zeugodacus caudatus	KT625492	678	162	1531	690	789	1137	940	1002	354	1341	297	1720	525	11166
Zeugodacus depressus	NC_071732	678	162	1534	690	789	1137	937	1023	354	1341	297	1720	525	11187
